# 3D-printed magnetic scaffolds promote bone and vessel regeneration through CRYAB/PI3K-AKT and NF-κB pathways identified by proteomics

**DOI:** 10.1016/j.bioactmat.2025.10.013

**Published:** 2025-10-23

**Authors:** Jieying Liu, Fuze Liu, Cairong Li, Zhengyao Li, Tianle Li, Yuanhao Wu, Di Wu, Yue Huang, Hui Chen, Hai Wang, Yuxiao Lai, Zhihong Wu

**Affiliations:** a Department of Orthopaedic Surgery, Peking Union Medical College Hospital, Peking Union Medical College and Chinese Academy of Medical Sciences, Beijing, 100730, China; b Centre for Translational Medicine Research & Development, Shenzhen Institutes of Advanced Technology, Chinese Academy of Sciences, China; c Center for Biomarker Discovery and Validation, National Infrastructures for Translational Medicine (PUMCH), Institute of Clinical Medicine, Peking Union Medical College Hospital, Chinese Academy of Medical Sciences & Peking Union Medical College, Beijing, 100730, China; d Plastic Surgery Hospital, Chinese Academy of Medical Sciences and Peking Union Medical College, No. 33 Badachu Road Shijingshan District, Beijing, China; e Faculty of Dentistry, University of Hong Kong, 34 Hospital Road, Prince Philip Dental Hospital, Sai Ying Pun, HKSAR, China; f Department of Orthopedic Surgery, Shanghai Sixth People's Hospital Affiliated to Shanghai Jiao Tong University School of Medicine, No.600 Yishan Road, Shanghai, 200233, China; g State Key Laboratory of Complex Severe and Rare Diseases, Peking Union Medical College Hospital, Peking Union Medical College and Chinese Academy of Medical Sciences, Beijing, 100730, China; h Stem Cell Facility, Institute of Clinical Medicine, Peking Union Medical College Hospital, Peking Union Medical College and Chinese Academy of Medical Sciences, Beijing, 100730, China; i Guangdong Engineering Laboratory of Biomaterials Additive Manufacturing, China

**Keywords:** 3D printing, Magnetic scaffolds, Osteogenesis, Angiogenesis, Proteomics

## Abstract

**Background:**

Iron oxide nanoparticles (IONPs)-based bone scaffolds have attracted increasing attention because of their potential to enhance osteogenesis and angiogenesis. However, the underlying mechanisms remain incompletely understood.

**Methods:**

We fabricated a biocompatible bone scaffold by incorporating γ-Fe_2_O_3_ magnetic nanoparticles into a PLGA matrix using 3D printing technology. The biosafety and effectiveness of the scaffold was validated through *in vitro* cell assays and *in vivo* implantation studies. To evaluate osteogenesis and neovascularization, we employed micro-CT imaging with a vascular contrast agent. In-depth mechanistic investigations were conducted via label-free proteomic profiling and pathway enrichment analysis.

**Results:**

The PLGA/Fe_2_O_3_ scaffolds demonstrated excellent biocompatibility and promoted both bone formation and angiogenesis *in vitro* and *in vivo*. Micro-CT analysis revealed enhanced new bone and vessel formation in the presence of magnetic scaffolds. Proteomic analysis revealed that alpha-B crystallin (CRYAB) is a key regulatory protein upregulated under a static magnetic field, thereby activating the PI3K/AKT signaling cascade and promoting osteogenic differentiation. In endothelial cells, we observed the upregulation of nuclear NF-κB and HIF-1α, leading to VEGF expression and angiogenic activation.

**Conclusion:**

Our findings provide direct evidence that 3D-printed PLGA/Fe_2_O_3_ scaffolds promote osteogenesis and angiogenesis both *in vitro* and *in vivo*. Importantly, we report for the first time that CRYAB-mediated stabilization of β-catenin plays a central role in magnetic scaffold-induced bone regeneration, offering new insights into the design of functional bone substitutes.

## Highlights

• **3D-Printed Magnetic Scaffolds**: This study demonstrates the development of biocompatible PLGA/Fe_2_O_3_ scaffolds using 3D printing technology to enhance both **osteogenesis** and **angiogenesis.**
• **Multi-Model Evaluation**: A comprehensive approach involving both **in vitro** and **in vivo** models, coupled with **Micro-CT imaging**, demonstrates enhanced **bone formation** and **vascularization**, showcasing the scaffolds' regenerative potential.
• **Mechanistic Insights via Proteomics**: The novel use of **label-free proteomic profiling** uncovers the central role of **alpha-B crystallin (CRYAB)** in stabilizing β-catenin, activating the **PI3K-AKT** and **NF-κB** signaling pathways to promote bone and vessel regeneration.

## Graphical abstract

**Figure:**
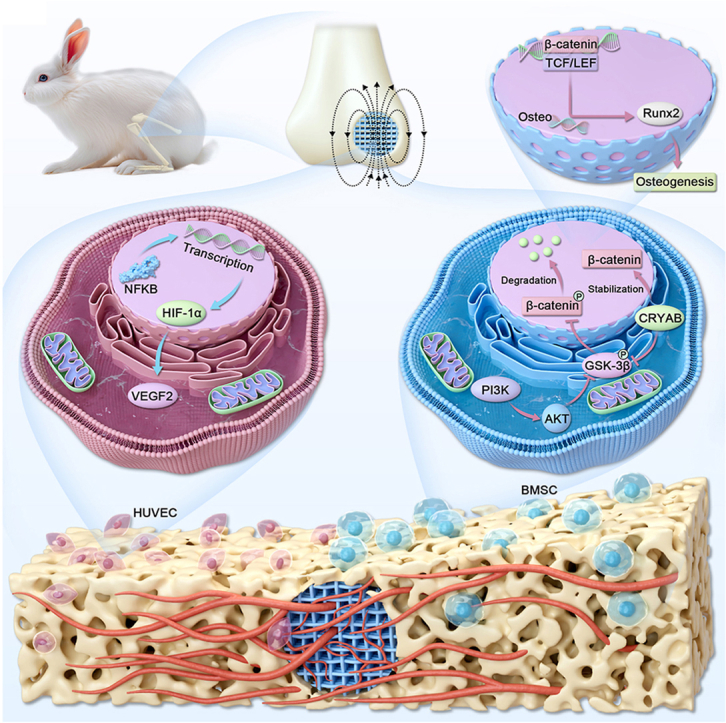

## 1 Introduction

Bone defects caused by trauma, tumors, or infection remain a significant challenge in clinical practice. These defects often lead to severe complications, such as delayed union and musculoskeletal dysfunction, profoundly impacting patients' quality of life [1,2]. Current therapeutic strategies primarily include autologous transplantation, allogeneic transplantation, and artificial scaffolds [3]. However, these approaches are associated with notable limitations. Autologous and allogeneic grafts are constrained by limited supply, donor-site morbidity, and immune rejection, but the complex and irregular geometries of bone defects further complicate their application [4]. Advancements in bone tissue engineering (BTE) have introduced 3D-printed scaffolds as a promising solution to accelerate bone regeneration. Unlike traditional scaffold fabrication methods, such as freeze-drying and solvent casting, which result in random internal architectures, 3D printing enables precise customization of internal structures to match the defect site. This tailored design significantly enhances the ability of the scaffold to support cell growth and facilitate bone healing [5,6].

Angiogenesis plays a crucial role in bone regeneration by promoting osteoblast activity and supporting the concurrent development of the vascular system and mineralized matrix [7,8]. In the absence of effective angiogenesis, vascular infiltration occurs too slowly, resulting in insufficient oxygen and nutrient supply for cell survival and bone tissue formation [9,10]. Therefore, strategies that integrate scaffolds, cells, and signaling molecules are essential for simultaneously enhancing osteogenesis and angiogenesis, thereby improving the efficacy of bone regeneration.

The incorporation of materials such as calcium phosphates, magnesium-based compounds, and gelatin methacrylate (GelMA), in addition to bioactive components such as growth factors and stem cells, markedly enhances the osteoinductive and angiogenic capabilities of 3D-printed scaffolds [11,12]. To address this, recent attention has turned toward introducing multifunctional nanomaterials that can simultaneously modulate cellular behavior and provide external field responsiveness. Among them, iron oxide nanoparticles (IONPs)-typically composed of iron, cobalt, or their alloys-have attracted considerable interest. IONPs are FDA-approved for applications such as MRI contrast enhancement, iron replacement therapy, and localized tumor hyperthermia [13]. Owing to their superparamagnetic properties, IONPs can be directed to specific tissues under an external magnetic field and demagnetized upon field removal, enabling precise spatial control [14,15]. Additionally, their excellent biocompatibility, ease of synthesis, nanoscale size, and low toxicity endow them with great potential in bone tissue engineering (BTE) [[16], [17], [18]]. Notably, IONPs have been shown to promote both osteogenesis and angiogenesis, even in the absence of an external magnetic stimulus [19,20]. Moreover, static magnetic fields (SMFs) have been reported to enhance bone regeneration by modulating cell membrane diamagnetism and ion channel flux [[21], [22], [23]].

Poly(lactic-co-glycolic acid) (PLGA), an FDA-approved biodegradable polymer, offers advantages such as tunable degradation rates, biocompatibility, and customizable microstructures through 3D printing [24,25]. Its degradation products are nontoxic, and its hydrophilicity can be tailored by adjusting the lactic/glycolic acid ratio [24].

In our previous work, we established optimal SMF conditions for IONPs promoting cell proliferation [26,27]. Building on these findings, the aims of this study were to (i) fabricate 3D printed IONPs-loaded PLGA scaffolds and evaluate their capacity to promote osteogenesis and angiogenesis under SMF both *in vitro* and *in vivo* and (ii) explore the underlying molecular mechanisms via bioinformatics analysis and validation through western blotting.

## 2 Materials and methods

### 2.1 Preparation of the PLGA/Fe_2_O_3_ scaffolds

The Fe_2_O_3_ magnetic nanoparticles were obtained from Aladdin (Shanghai, China), and PLGA was purchased from the Shandong Institute of Medical Instruments (Jinan, China). 1,4-Dioxane was obtained from Shanghai LingFeng Chemical Reagent Co., Ltd. (Shanghai, China). PLGA was dissolved in 1,4-dioxane at a concentration of 15 g per 100 mL and stirred overnight. Then Fe_2_O_3_ magnetic nanoparticles were dispersed into the PLGA solution with the content of 0 %, 10 %, 20 % and 30 % (PLGA, P10F, P20F, P30F). And ultrasonic shaking was used to disperse nanoparticles evenly. The solution of PLGA containing Fe_2_O_3_ were spurted out layer by layer at the thickness of 200 μm by low-temperature 3D printing at −30 °C(CLRF-2000- II, Tsinghua University, Beijing, China). During the 3D printing process, the nozzle diameter is 200 μm and the temperature was set to 12 °C, the scanning rate was 22 mm s^−1^, the spinneret filling rate was 0.3 mm s^−1^,the thickness of the support layer is set to 400 mm. Finally, a cubic porous scaffold was fabricated under the low temperature of −30 °C. Following preparation, scaffolds were freeze-dried in a freeze dryer (Alpha 2–4 LDplus, Marin Christ, Germany) for 48h and then dried in a vacuum oven at 37 °C for 7 days to completely remove solvent 1,4-dioxane [28]. The obtained scaffolds were referred to as PLGA, P10F and P20F. Scanning electron microscopy (SEM, Zeiss Supra55, Carl Zeiss, Germany) and energy dispersive spectroscopy (Oxford X-Max 20) coupled with SEM were used to observe the surface morphology, pore size and elemental composition of the three groups of scaffolds. The porosity was quantitatively evaluated using the ethanol displacement method. In brief, specimens (10 × 10 × 10 mm^3^) were prepared, and their apparent volume (V_1_) was determined through geometric measurement. The scaffolds were subsequently fully immersed in a graduated cylinder containing a known volume of ethanol (V_2_). The system was subjected to vacuum treatment until bubble formation ceased, with the final liquid level recorded as V_3_. The porosity was calculated using the following equation: Porosity (%)=(V_1_+V_2_-V_3_)/V_1_ × 100 %.

### 2.2 Physicochemical properties of the scaffolds

#### 2.2.1 Nanoparticle tracking analysis (NTA)

Nanoparticle size and concentration were measured using a ZetaView® particle tracking analyzer (PMX-110, Particle Metrix) with a 488-nm laser. The system was pre-rinsed with 0.22 μm-filtered buffer and calibrated using 100 nm polystyrene standards before each run.

IONPs dispersions and extracts from PLGA and P20F scaffolds (in pH 7.4 PBS buffer at 37 °C, for 6 and 8 weeks) and the supernatant was analyzed. Z-stack videos were acquired at 11 cell positions (60–90 s per position, 25–30 fps) under constant settings (camera sensitivity 80, shutter 100, minimum area 10, minimum brightness 30). Data were processed using ZetaView software, with particle size distribution and concentration reported as mean ± SD from three independent measurements.

#### 2.2.2 Water contact angle (WCA) test

The water contact angles of the three scaffold groups (PLGA, P10F, and P20F) were determined using a contact angle goniometer (Drop Shape Analyzer, KRUSS, Germany) to assess alterations in the surface hydrophilicity of the different scaffolds (n = 5).

#### 2.2.3 Mechanical test

PLGA/Fe_2_O_3_ porous scaffolds (10 × 10 × 10 mm^3^) were sectioned for compressive mechanical characterization using a universal testing system (ElectroPuls E10000, Instron, USA). The compression performance of the scaffold in the vertical and horizontal directions was tested at a compression speed of 2 mm/min. Five samples were tested for each sample type (n = 5).

#### 2.2.4 Degradation performance

The scaffolds were immersed in PBS buffer (pH 7.4) at 37 °C at a volume ratio of 0.1 g mL^−1^, and their supernatants were collected every week for 8 weeks. The pH of the collected solution at each time point was measured by a pH meter (Mettler-Toledo, USA). The concentration of Fe^3+^ released from the scaffold was determined by ICP-OES (700 series, Agilent Technologies, USA) at 2, 4, 6, and 8 weeks.

### 2.3 Magnetic stimulation

In the *in vitro* experiments, the optimum magnetic conditions for stimulating cells were adapted from our previous studies [5,20,26]. To generate a static magnetic field, a neodymium-iron-boron (NdFeB) permanent magnet was placed beneath the cell culture plates. The distance between the magnet and the cells was maintained at approximately 1 cm to ensure a field strength of ∼100 mT at the cell level. All groups, including the PLGA scaffolds, were subjected to the same magnetic field conditions to maintain consistency across the experimental settings (Fig. 3C and D). To provide continuous SMF exposure *in vivo*, NdFeB permanent magnets were arranged evenly beneath the bottom plate of the rabbit cage to generate a uniform static magnetic field covering the entire activity area (Fig. 4A). The magnets were fixed to ensure a constant distance between the source and the surgical site, minimizing field fluctuation during the exposure period. This configuration allowed rabbits to move freely while receiving continuous magnetic stimulation in a reproducible manner. This layout is consistent with magnetic field application models in wound healing and tissue regeneration studies, where multiple magnets are placed around the target site to achieve localized and uniform SMF exposure throughout the experimental period [5].

### 2.4 Cell culture

Human BMSCs were obtained from Cyagen Biosciences (Cyagen Biosciences, Santa Clara, CA, USA) and cultured in MSC medium (Cyagen Biosciences) supplemented with 10 % fetal bovine serum (FBS) and 1 % penicillin/streptomycin at 37 °C and 5 % CO_2_. BMSCs at passages three to five were used for the *in vitro* studies. Human umbilical vein endothelial cells (HUVECs) were obtained from the Chinese Academy of Medical Sciences (Beijing, China) and cultured in high-glucose Dulbecco's modified Eagle's medium (DMEM; Gibco BRL, Grand Island, USA) supplemented with 10 % FBS and 1 % penicillin‒streptomycin. When they reached approximately 90 % confluence, the cells were passaged every 2–3 days. All scaffolds were sterilized by immersion in 75 % ethanol for 48 h, followed by UV irradiation for 30 min on each side. For CCK-8 viability and ALP activity assays, sterilized scaffolds (2.5 × 2.5 × 1 mm^3^) were placed in 48-well plates seeded with cells and co-cultured under standard conditions. For Alizarin Red staining, transwell migration, and scratch wound assays, larger scaffolds (5 × 5 × 1 mm^3^) were placed in 24-well plates with pre-seeded cells and co-cultured for the indicated durations. The same batch of scaffolds was used across replicates within each experiment to ensure consistency.

### 2.5 Cell proliferation assay

A proliferation assay of the BMSCs and HUVECs was performed to explore the biocompatibility of the IONPs-loaded scaffolds with a cell counting kit-8 (CCK-8; Dojindo, Tokyo, Japan) assay. Scaffolds were co-cultured with BMSCs and HUVECs. After the cells were cultured for 1, 3, 5, or 7 days, the culture medium was removed, and fresh culture medium supplemented with 10 % CCK-8 solution was added to each group. Afterward, the groups were incubated for 1 h at 37 °C. The absorbance was measured at 450 nm with a microplate reader (Bio-Rad Laboratories, Inc., Hercules, CA, USA), and the optical density was used to determine cell viability.

Live/dead staining was also conducted for the cell variability assay. After 48 h of incubation, the cell-seeded scaffolds were carefully retrieved from the 24-well plates. Each scaffold was gently rinsed three times with phosphate-buffered saline (PBS) to remove residual culture medium. A total of 200 μL of staining solution-prepared by mixing 2.5 μL of calcein-AM and 10 μL of ethidium homodimer-was immediately added to each scaffold. The samples were incubated in the dark at 37 °C for 20 min to allow for live/dead staining. Following incubation, the entire scaffold was imaged using a fluorescence microscope (nikon A1). For 3D reconstruction, Z-stack images covering the full thickness of the scaffold were acquired using a confocal laser scanning microscope. The images were subsequently processed and reconstructed into a 3D projection using NIS-Elements AR (Nikon Instruments), allowing visualization of the spatial distribution of viable and dead cells within the scaffold. The distribution and proportion of viable cells were analyzed on the basis of the fluorescence signals.

### 2.6 Cell migration and transwell assays

To investigate the effect of the PLGA/Fe_2_O_3_ scaffolds on HUVEC migration, two complementary assays were performed under static magnetic field conditions. All the experimental groups were exposed to SMF throughout the procedures.

**Scratch (Wound Healing) Assay:** HUVECs were seeded at a density of 1 × 10^5^ cells per well into 24-well plates and cultured until they reached 80–90 % confluence. A straight scratch was made in the monolayer using a 200 μL pipette tip, ensuring a consistent scratch width across all the wells. Scaffolds from each group (Blank, PLGA, P10F, P20F) were then gently placed on the scratched area (Fig. 3D). After 24 h of incubation, the scaffolds were carefully removed, and the wound closure area was photographed using a phase-contrast microscope.

**Transwell Migration Assay:** Transwell inserts (8-μm pore size; 24-well format; Corning, NY, USA) were precoated with Matrigel (BD Biosciences, USA) and allowed to solidify at 37 °C. HUVECs (1 × 10^4^ cells/well) were suspended in serum-free medium and seeded in the upper chamber. The lower chamber was filled with medium containing 10 % FBS as a chemoattractant, and the scaffolds were placed at the bottom of the lower chambers. After 48 h of incubation under SMF (Fig. 3C), nonmigrated cells on the upper surface were gently removed with a cotton swab. The cells that had migrated to the lower surface were fixed with anhydrous ethanol, stained with 0.1 % crystal violet (Beyotime, Shanghai, China), and imaged under a light microscope (Leica, Solms, Germany). The number of migrated cells was quantified using ImageJ software.

### 2.7 Tube formation assay

A tube formation assay was conducted to evaluate the proangiogenic potential of the scaffold extract on HUVECs. Matrigel (356234, BD Biosciences, San Jose, USA) was thawed overnight at 4 °C and added to 24-well plates at a volume of 200 μl per well. The plates were incubated at 37 °C for 1 h to allow for the Matrigel to polymerize and form a gel-like basement membrane.

HUVECs were harvested and resuspended at a density of 2 × 10^4^ cells per well in complete medium supplemented with 10 % FBS. After the Matrigel solidified, the medium was replaced with 48-h extracts of different scaffolds (blank, PLGA, P10F, and P20F), which were prepared by immersing each scaffold type in culture medium at 37 °C. The scaffold extracts were used to replace the culture supernatant in each group.

Cells were then seeded onto the Matrigel-coated wells and incubated at 37 °C for 6 h. Tube-like structures were observed using an inverted phase-contrast microscope, and images were captured for analysis. Tube formation was quantified using ImageJ software (Media Cybernetics, Bethesda, USA), and parameters such as the total tube length were measured.

### 2.8 Osteogenic differentiation

To evaluate the osteogenic differentiation induced by the PLGA/Fe_2_O_3_ scaffolds, BMSCs were first seeded onto plates and allowed to adhere overnight. Once the cell monolayer was established, the scaffolds from different groups (Blank, PLGA, P10F, P20F) were gently placed on top of the cell layer in each well. All groups were maintained under a static magnetic field (SMF) for the entire induction period.

The standard culture medium was then replaced with osteogenic induction medium (Cyagen Biosciences). The medium was changed every three days. On Day 14, the scaffolds were carefully removed, and the remaining cell layers were washed twice with PBS. The cells were fixed with 4 % paraformaldehyde for 15 min and stained with 2 % Alizarin Red S (pH 4.2) at room temperature for 20 min to assess calcium deposition. After staining, the wells were thoroughly washed with deionized water, and images were acquired using a light microscope.

Additionally, alkaline phosphatase (ALP) activity—an early osteogenic marker—was quantified on Days 7 and 14 using a pNPP-based ALP assay kit (BioAssay Systems, Biocore, NSW, Australia). Cells were lysed in 100 μl of 1 % NP-40 buffer, and ALP activity was measured according to the manufacturer's instructions. The total protein content was determined using a BCA protein assay kit (Thermo Scientific, Waltham, MA, USA), and ALP activity was expressed as the enzyme activity per milligram of protein (U/mg).

### 2.9 Animal model and micro-CT analysis

All the animal experiments were approved by the ethics committee of the Peking Union Medical College Hospital (Y0638). The experiments were conducted in accordance with the Guide for the Care and Use of Laboratory Animals (GB14925-2010; NIH) and the Laboratory Animal Center of Peking Union Medical College Hospital.

**Bone defect model establishment:** All animals were randomly assigned to experimental groups using a random number generator prior to surgery. New Zealand rabbits were anesthetized with an intraperitoneal injection of pentobarbital sodium 50 mg/kg isoflurane. The bilateral skin was incised, the muscles were bluntly dissected, the distal femoral condyle was exposed, and tunnels 10 mm in diameter and 1.5 cm in depth were drilled in it with an electric drill. Appropriate scaffolding was implanted into the tunnel. Three groups of subjects were randomly selected: control, PLGA, and P20F (n = 6 for each time point). Following surgery, the experimental rabbits were injected intraperitoneally with penicillin (100 000 UI penicillin solution/kg body weight) to prevent infection. Experimental rabbits were anesthetized with isoflurane gas at 6 and 12 weeks after surgery. Femoral condyles were isolated for micro-CT and histological analysis.

**CT-based bone analysis:** To assess bone regeneration, spinal samples (n = 6 per group) were fixed in 4 % phosphate-buffered formaldehyde for 24 h and stored in 70 % ethanol until scanning. The samples were imaged using a high-resolution micro-CT system (μCT-40; Scanco Medical, Bassersdorf, Switzerland) with a voxel size of 36 μm, an X-ray tube voltage of 70 kV, and a current of 114 μA. Axial projections were reconstructed into cross-sectional images using the manufacturer's reconstruction software (Scanco μCT Evaluation Program V6.5-3). The samples were subsequently scanned using a high-resolution micro-CT system (μCT-40, Scanco Medical, Bassersdorf, Switzerland) with a voxel size of 36 μm. Cross-sectional scans were acquired, and three-dimensional reconstruction was performed using standardized parameters (sigma = 1.2, support = 1.0, threshold = 158 mg/cm^3^). The reconstructed images were generated using the manufacturer's software to evaluate osseous tissue fusion across the defect site between adjacent spinous processes.

The region of interest (ROI) encompassed the entire defect zone between adjacent spinous processes. Three-dimensional reconstructions were generated from 2D slices, and bone morphometric parameters were calculated according to standard guidelines. These included: **BV/TV** (bone volume fraction): ratio of segmented bone volume to the total ROI volume (%); **Tb.Th** (trabecular thickness): mean thickness of trabecular structures within the ROI, computed using direct 3D distance transformation (mm); **Tb.N** (trabecular number): number of trabecular structures per mm, calculated as (BV/TV)/Tb.Th; **Tb.Sp** (trabecular separation): mean distance between trabeculae, computed as the inverse of Tb.N minus Tb.Th; **BMD** (Bone Mineral Density): Determined by calibration with manufacturer-supplied hydroxyapatite phantoms (mg HA/cm^3^)

**CT-based angiography:** To visualize neovascularization, CT-based angiography was performed (n = 6 per group). After euthanasia, the abdominal aorta was systemically perfused with heparinized saline (100 U/mL) under physiological pressure, followed by perfusion with 10 % neutral-buffered formalin to fix the vasculature. A radiopaque silicone rubber casting agent, Microfil 117 (Flow Tech Inc., South Windsor, CT), was injected and allowed to fully polymerize at 4 °C for 24 h. Immediately before use, Microfil was prepared by mixing MV-Diluent: MV-117(orange compound):MV-Curing Agent at 38:47.5:4.5 (vol/vol/vol), per widely used protocols for vascular μCT casting; the mixture was gently degassed prior to perfusion [29]. After they were cured, the spinal segments containing the scaffold were harvested, fixed in 10 % neutral-buffered formalin for 48 h at 4 °C, and dissected to isolate the bone surrounding the defect.

The samples were decalcified in 9 % formic acid until radiolucent. Decalcified samples were scanned using a high-resolution micro-CT system (μCT-40; Scanco Medical) at isotropic voxel sizes of 10.5 μm, 70 kV, and 114 μA.Image reconstruction and vascular segmentation were performed using the Scanco μCT Evaluation Program V6.5-3. A global thresholding method was applied with a grayscale threshold of 130 (arbitrary units) to segment the perfused radiopaque Microfil® from surrounding tissues. A 10.13039/100014230Gaussian filter (sigma = 1.2, support = 1.0) was applied to reduce noise. The ROI was defined as the defect area containing the scaffold. Three-dimensional vascular reconstructions were generated, and the vascular parameters were calculated as follows: **Vascular volume**: ratio of the vessel volume to the total ROI volume (%). All image segmentation and quantification were performed using identical thresholds and by investigators blinded to group allocation.

### 2.10 Histological analysis

The collected specimens were fixed in 10 % paraformaldehyde solution, decalcified with 5 % EDTA (Boster Biological Technology Co., Ltd., AR1071) and embedded in paraffin. Each selection was 5-μm thick in this experiment. Goldner's trichrome staining and immunohistochemical staining were performed. Goldner's trichrome staining (Sigma‒Aldrich, HT10316) was conducted according to the instructions, and the sections were stained with hematoxylin (Sigma‒Aldrich, 03971) for 20 min and then rinsed with ethanol. The sections were immersed in xylene and sealed with resin. For immunohistochemical analysis, the sections were rehydrated, blocked, and incubated with primary anti-OCN (1:100), anti-CD31 (1:100), and anti-VEGFA (1:100) antibodies overnight at 4 °C. Afterward, the membranes were incubated with secondary antibodies at room temperature for 1 h. Histological sections were coded by an independent technician, and scoring/quantification was carried out by two independent observers.

### 2.11 Proteomic and bioinformatics analysis

Proteomic Analysis: After the hBMSCs and HUVECs were seeded, the scaffolds (Blank, PLGA and P20F, n = 3) were carefully placed onto the cell layer and cocultured for 48 h. Proteomic analysis was subsequently performed. Label-free quantification and protein identification were used to explore the differences in proteomic profiles between the groups. Total proteins from hBMSCs were extracted using 4 % SDS lysis buffer and incubated at 20 °C for 30 min. Protein purification and digestion were carried out using a filter-aided sample preparation (FASP) protocol. After enzymatic digestion with trypsin, the resulting peptides were purified and desalted using C18 solid-phase extraction columns (Sep-Pak, Waters, Milford, MA, USA) operated with a Qiavac 24 Plus vacuum manifold (Qiagen, Germantown, MD, USA). Mass spectrometry analysis was conducted on a Q Exactive mass spectrometer (Thermo Fisher Scientific) coupled with an Easy-nLC 1000 system (Thermo Fisher Scientific). The raw data were processed and analyzed using MaxQuant software (v1.5.3.8; Max Planck Institute of Biochemistry, Planegg, Germany). For proteomic data analysis, peptide and protein identification was performed using a target–decoy search strategy with a false discovery rate (FDR) controlled at < 1 % at both the peptide-spectrum match and protein levels. For differential expression analysis, we calculated p-values using a two-sided *t*-test. To facilitate biological interpretation and avoid over-stringent filtering, proteins with p < 0.05 were selected as differentially expressed proteins (DEPs) for downstream GO and KEGG enrichment analyses. To provide transparency, we additionally computed Benjamini–Hochberg (BH) adjusted q-values for all quantified proteins, which are included in Supplementary Table 1. This allows readers to apply more stringent cut-offs (e.g., q < 0.05) if desired.

Differentially expressed proteins (p < 0.05 and fold change (FC) > 1.5 or < 1/1.5) between the scaffold-stimulated and control groups were subjected to Gene Ontology (GO) enrichment and Kyoto Encyclopedia of Genes and Genomes (KEGG) pathway analyses. The identified proteins were classified into biological process, cellular component, and molecular function categories on the basis of their GO annotations. The R software package limma was used to analyze the differentially expressed genes. For key target genes related to angiogenesis and osteogenesis, Kyoto Encyclopedia of Genes and Genomes (KEGG) pathway enrichment analysis was performed. We visualized the top 20 most significantly enriched GO terms and KEGG pathways.

### 2.12 Western blotting

A BCA protein assay kit (Thermo Scientific, Waltham, MA, USA) was used to measure the concentration of proteins, which were isolated with RIPA lysis solution (Sigma). SDS‒PAGE (sodium dodecyl sulfate‒polyacrylamide gel electrophoresis) gels were used to separate the proteins on the basis of molecular weight. The proteins were transferred to PVDF membranes (Millipore Corporation, Billerica, MA, USA) and blocked with 5 % nonfat milk in Tris-buffered saline containing 0.1 % Tween 20 (TBST) for 2 h at room temperature. Afterward, the membranes were incubated with primary antibody at 4 °C overnight, followed by incubation with HRP-linked secondary antibodies for 1 h at room temperature. The protein bands were visualized using an enhanced chemiluminescence detection system (SuperSignal West Pico, Thermo Scientific) and analyzed with ImageJ. All protein expression levels were normalized to those of the internal controls, GAPDH and vinculin.

**Co-immunoprecipitation (Co-IP) Assay.** To verify the interaction between CRYAB and β-catenin, co-immunoprecipitation (Co-IP) was performed. Briefly, BMSCs were cultured and stimulated with PLGA or P20F scaffolds for 24 h. Total protein lysates were collected and incubated with anti-CRYAB antibody overnight at 4 °C, followed by immunoprecipitation using a Thermo Scientific™ Pierce™ Magnetic IP/Co-IP Kit according to the manufacturer's protocol. The immunoprecipitated complexes were subjected to SDS-PAGE and Western blotting. Immunology complexes were analyzed with anti-CRYAB and anti-β-catenin antibodies to quantify the amount of β-catenin associated with CRYAB under different stimulation conditions.

### 2.13 Statistical analysis

All experiments were repeated at least three times per group. Statistical analysis was conducted with GraphPad Prism 7 software (La Jolla, CA, USA). Quantitative data from these experiments are shown as the means ± standard deviations (SDs). Student's *t*-test was used for comparisons between two groups, and multiple groups were compared by two-way analysis of variance with Tukey's post hoc test. Statistical significance was defined as p < 0.05.

Data are representative of these experiments and are shown as the means ± standard deviation (SDs). The two treatment groups were compared by Student's *t*-test. Multiple group comparisons were performed. Statistical analysis was performed using GraphPad Prism 7.0 software, with statistical significance declared at (∗) p < 0.05, (∗) p < 0.01, and (∗) p < 0.001.

## 3 Results

### 3.1 Fabrication and characterization of the scaffolds

**Fig. 1:**
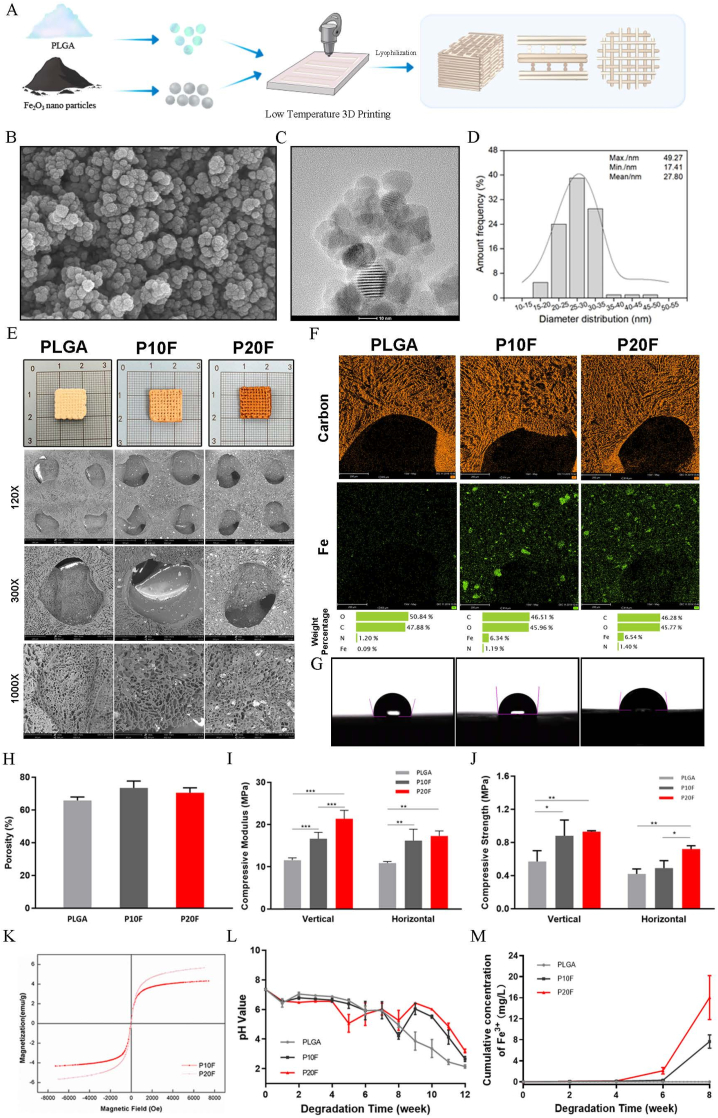
**Fabrication and characterization of PLGA/Fe_2_O_3_ composite scaffolds.** (A) Schematic illustration of scaffold fabrication via low-temperature 3D printing. PLGA was mixed with Fe_2_O_3_ nanoparticles at different ratios (0 %, 10 %, and 20 %), followed by printing and lyophilization to generate porous bone scaffolds. (B) SEM image and (C) TEM image of Fe₂O₃ nanoparticles showing uniform spherical morphology and distinct lattice fringes. (D) Particle size distribution of Fe₂O₃ nanoparticles with an average diameter of ∼27.8 nm. (E) Macroscopic and SEM characterization. Top row: photographic images of PLGA, PLGA/10 %Fe_2_O_3_ (P10F), and PLGA/20 %Fe_2_O_3_ (P20F) scaffolds. Bottom row: SEM images at 120 × , 300 × and 1000 × showing the cross-sectional structure and pore morphology. (F) Energy-dispersive X-ray spectroscopy (EDS) mapping analysis showing the elemental distributions of carbon and iron. The quantitative elemental composition is shown in the bottom panel. (G) Water contact angle measurements indicating changes in scaffold hydrophilicity with increasing Fe_2_O_3_ content. (H) Porosity analysis showing comparable porosity across groups. (I, J) Mechanical testing of the compressive modulus (I) and compressive strength (J) in the vertical and horizontal directions. Fe_2_O_3_ incorporation enhanced mechanical performance. (K) Magnetization curves (M − H loops) demonstrating the superparamagnetic behavior of the P10F and P20F scaffolds. (L) pH variation during scaffold degradation over 12 weeks, indicating mild acidity changes. (M) Cumulative release profile of Fe ions from the scaffolds over 8 weeks, with P20F showing a higher but stable release rate.

In this study, Fe_2_O_3_ magnetic nanoparticles were incorporated into a 1.4-dioxane solution with 15 wt% PLGA at concentrations of 0 wt%, 10 wt%, and 20 wt%. The composite solutions were then fabricated via low-temperature extrusion-based 3D printing following the predefined architecture shown in Fig. 1A, with the freeze-dried scaffolds subsequently designated as PLGA, P10F, and P20F. As shown in the SEM image (Fig. 1B), TEM image (Fig. 1C), and particle size distribution curve (Fig. 1D), the Fe_2_O_3_ particles exhibit uniform size. Approximately 90 % of the particles have a size ranging from 20 to 35 nm, with an average size of about 27.8 nm. The macroscopic photographs, SEM micrographs(Fig. 1E), and corresponding elemental maps (Carbon: orange, Fe: green) of the PLGA, P10F, and P20F scaffolds are shown in Fig. 1F. The scaffolds exhibited a hierarchical porous architecture, featuring interconnected macropores (200–400 μm) generated through 3D printing, superimposed with micropores (5–50 μm) formed via phase separation. The porous structure is beneficial for cell adhesion and nutrient transport and promotes osteoconductivity at the implantation site. The water contact angles (WCAs) of the scaffolds were determined. The surface WCA of Fe_2_O_3_/PLGA decreased with increasing Fe_2_O_3_ content, while the WCA of P20F was less than 90°, indicating that it was hydrophilic (Fig. 1G), which is beneficial for cell adhesion.

The porosity of the scaffold tested by ethanol extraction was ≈80 %, and there was no significant difference between the PLGA group and the magnetic-containing particle group, as shown in Fig. 1H. The mechanical properties of the scaffolds in the vertical and horizontal directions were tested, and the results are shown in Fig. 1I and J. The compressive modulus and strength of the Fe_2_O_3_/PLGA scaffolds increased with increasing Fe_2_O_3_ particle content. As shown in Fig. 1I, when the magnetic particle content reached 20 wt%, the vertical and horizontal compressive moduli of the PLGA/Fe_2_O_3_ scaffolds were approximately 21.37 ± 2.00 MPa and 17.29 ± 1.19 MPa, respectively. The Fe_2_O_3_ magnetic nanoparticles exhibited superparamagnetic properties. The saturation magnetization was 4.32 emu/g and 5.66 emu/g in P10F and P20F, respectively, and the superparamagnetic properties of the scaffolds increased with increasing nanoparticle content, as shown in Fig. 1K.

The degradation performance of the PLGA/Fe_2_O_3_ scaffolds was investigated in phosphate-buffered saline (PBS, pH 7.4) at 37 °C for 12 weeks. The pH of the degradation solution was recorded every week (Fig. 1L). The pH of the PLGA group did not significantly change until it declined instantaneously at 8 weeks because of the acidolysis of PLGA. The pH values of the P10F and P20F groups slightly increased at 8 weeks and 4 weeks. We found that this was caused by the degradation of Fe_2_O_3_ in P20F and P10F at 4 weeks and 8 weeks through the detection of the Fe^3+^ concentration in the degradation liquid. As shown in Fig. 1M, the Fe^3+^ in the P20F and P10F scaffolds began to degrade after 4 weeks, which is consistent with the results of the pH value. During the 4 weeks prior to the scaffold degradation experiment, no iron ions were detected, which was related to the solubility properties of Fe_2_O_3_. Fig. S2A shows that as the degradation process progresses, the mechanical properties of the composite scaffold decline. Whether it is 4 weeks or 8 weeks, the mechanical properties of P20F are significantly better than those of the other groups. Moreover, no significant difference was detected in the nanoparticle content of the extract solutions between PLGA and P20F at different time points (Fig. S2B).

### 3.2 Biocompatibility properties of the PLGA/Fe_2_O_3_ scaffolds

**Fig. 2:**
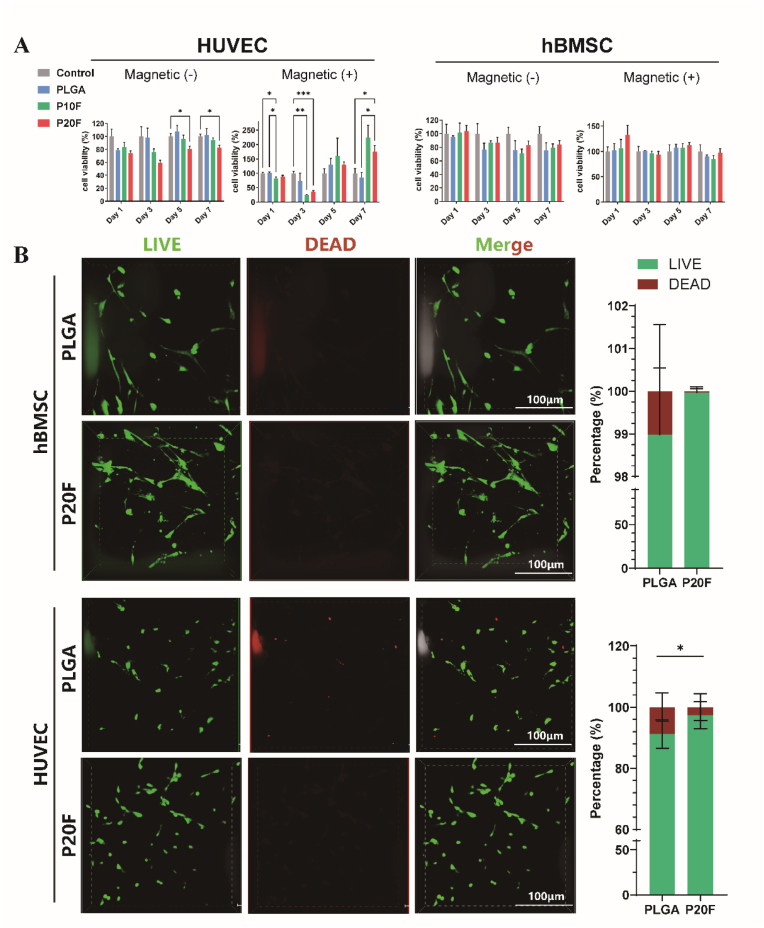
***In vitro* cytocompatibility of the PLGA/Fe_2_O_3_ scaffolds with and without magnetic field stimulation**. (A) Viability of HUVECs and BMSCs cultured on different scaffolds (PLGA, PLGA/10 %Fe_2_O_3_ [P10F], and PLGA/20 %Fe_2_O_3_ [P20F]) under magnetic field-free [Magnetic(−)] and magnetic field [Magnetic(+)] conditions. CCK-8 assays were performed on Days 1, 3, 5, and 7. The data are presented as the means ± SDs. Two-way ANOVA followed by Tukey's post-hoc test.∗P < 0.05, ∗∗P < 0.01, ∗∗∗P < 0.001. (B) Live/dead staining images of BMSCs and HUVECs cultured on PLGA and P20F scaffolds after 3 days. Green: live cells; red: dead cells. Quantitative analysis revealed a significantly greater percentage of viable cells on P20F scaffolds than on PLGA in both cell types. Scale bars: 100 μm. The data are presented as the means ± SDs. unpaired two-tailed Student's t-test ∗P < 0.05, ∗∗P < 0.01.

To evaluate the biocompatibility of the PLGA/Fe_2_O_3_ scaffolds, BMSCs and HUVECs were cocultured with the scaffolds, and cell viability was assessed at different time points (1, 3, 5, and 7 days) using a CCK-8 assay and live/dead staining. As shown in Fig. 2A, no significant differences in cell viability were observed among the scaffold groups, regardless of the presence or absence of a static magnetic field (SMF) in the BMSCs. Fig. S1C revealed that P20F and P30F scaffolds showed a significant increase in cell viability on Day 5 compared to the PLGA and blank groups (*p* < 0.05∗ or *p* < 0.01∗∗).

**3:**
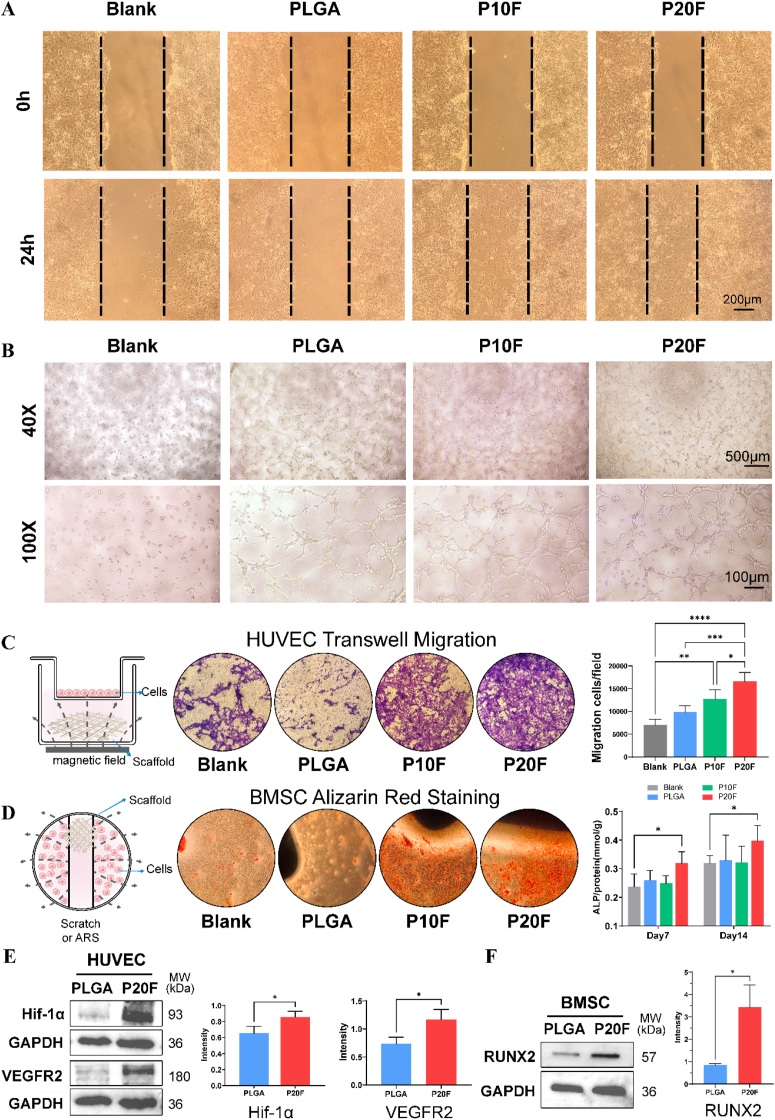
In vitro evaluation of the proangiogenic and osteogenic potential of PLGA/Fe2O3 scaffolds under magnetic field stimulation. (A) Scratch wound healing assay of HUVECs cultured with different scaffolds (blank, PLGA, PLGA/10 %Fe_2_O_3_ [P10F], and PLGA/20 %Fe_2_O_3_ [P20F]) under magnetic field stimulation (MF). Images at 0 h and 24 h show that the P20F + MF group exhibited the highest cell migration capacity. Scale bars: 200 μm (B) *In vitro* tube formation assay of HUVECs cultured with scaffold extracts under MF. Representative images at 40 × and 100 × magnification demonstrate enhanced tube-like structures. Scale bars: 500 or 100 μm (C) Transwell migration assay of HUVECs after 24 h of incubation with different scaffolds. Quantification revealed a significant increase in the number of migrated cells in the P20F group. The data are presented as the means ± SDs. Two-way ANOVA followed by Tukey's post-hoc test.∗P < 0.05, ∗∗P < 0.01, ∗∗∗P < 0.001. ∗∗∗∗P < 0.0001. (n ≥ 6) (D) Alizarin Red S staining of BMSCs cultured with different scaffolds for 14 days. The P20F group shows enhanced mineral deposition. Quantification of ALP activity at Days 7 and 14 supported the osteogenic trend. Two-way ANOVA followed by Tukey's post-hoc test.∗P < 0.05. (n ≥ 3) (E) Western blot analysis of HIF-1α and VEGFR2 expression in HUVECs after treatment with PLGA and P20F scaffolds under MF. Densitometric analysis revealed significant upregulation in the P20F group. GAPDH served as a loading control. unpaired two-tailed Student's t-test ∗P < 0.05, ∗∗P < 0.01. (n = 3) (F) Western blot of RUNX2 expression in BMSCs cultured with PLGA and P20F scaffolds. RUNX2 protein expression significantly increased in the P20F group. unpaired two-tailed Student's t-test ∗P < 0.05, ∗∗P < 0.01. (n = 3).

**Fig. 4:**
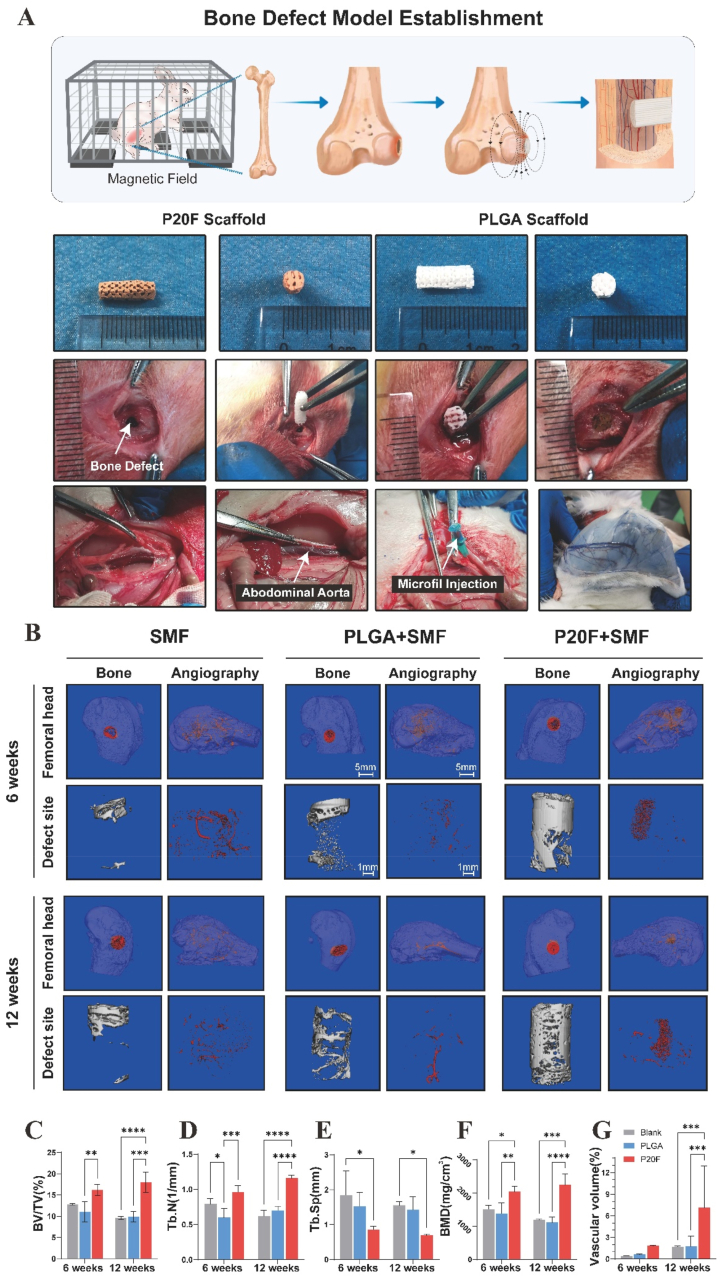
***In vivo* evaluation of bone regeneration and angiogenesis in a rabbit femoral defect model under static magnetic field (SMF) stimulation**. (A) Schematic and surgical procedure. Illustration showing the surgical design of a cylindrical critical-sized defect in the distal femur of rabbits, followed by scaffold implantation and SMF exposure. The images below display the morphology of different scaffolds (PLGA, 20 % IONPs, and control), and their corresponding implantation into the bone defect site. (B) Representative 3D micro-CT reconstructions of bone (left column) and vessels (angiography, right column) in the femoral head and defect region at 6 and 12 weeks post-implantation. Compared with the PLGA and SMF-only groups, the 20 % IONP scaffold under the SMF (P20F + SMF) group exhibited increased bone formation and neovascularization. (C–F) Quantitative analysis of bone volume at 6 and 12 weeks. Bone volume was significantly greater in the P20F group than in the PLGA and blank groups. The data are presented as the means ± SDs. Two-way ANOVA followed by Tukey's post-hoc test.∗P < 0.05, ∗∗P < 0.01, ∗∗∗P < 0.001. ∗∗∗∗P < 0.0001. (G) Quantitative analysis of angiographic volume at 6 and 12 weeks. Compared with the other groups, the P20F group exhibited markedly increased vessel volume. Two-way ANOVA followed by Tukey's post-hoc test.∗P < 0.05, ∗∗P < 0.01, ∗∗∗P < 0.001. ∗∗∗∗P < 0.0001.

In contrast, HUVECs exhibited a more dynamic response. On Day 3, a slight decrease in cell viability was observed in the SMF-treated groups, particularly in those cultured with IONPs-loaded scaffolds. This reduction may reflect a transient adaptation period in response to external magnetic stimulation. However, by Day 7, compared with the those in other groups, HUVECs in the SMF + IONPs scaffold group showed a marked increase in cell viability, suggesting that the synergistic effect of magnetic nanoparticles and a static magnetic field promoted endothelial cell growth. As shown in Fig. S1A, cell viability of HUVECs cultured on P10F, P20F, and P30F scaffolds was significantly increased compared with the PLGA and blank groups on Day 7 (*p* < 0.05∗ or *p* < 0.01∗∗).

LIVE/DEAD staining further confirmed the CCK-8 findings (Fig. 2B). Fluorescence images revealed a predominance of green-stained live cells with minimal red-stained dead cells in both BMSC and HUVEC cultures, indicating excellent cell survival on the scaffolds. The cell morphology remained spread and well attached. The 3D reconstruction further highlighted that cell penetration extended into the inner pores of the scaffold, suggesting favorable cell–scaffold interactions and a supportive microenvironment for tissue regeneration (Supplementary Video 1-4). Together, these results suggest that the scaffolds exhibit excellent biocompatibility and support the attachment, survival, and proliferation of both BMSCs and HUVECs.

### 3.3 The PLGA/Fe_2_O_3_ scaffold promotes angiogenesis in HUVECs

The proangiogenic effect of the PLGA/Fe_2_O_3_ scaffolds on HUVECs was evaluated using scratch wound healing, transwell migration and tube formation assays, as shown in Fig. 3A–C. After 24 h of coculture, compared with the blank and PLGA groups, the PLGA/Fe_2_O_3_ scaffold group exhibited significantly enhanced HUVEC wound healing capacity (Fig. 3A). Consistently, Transwell assays demonstrated a significant increase in the number of migrated HUVECs in both the P10F and P20F groups, with the P20F scaffold exhibiting superior migration-promoting effects over P10F (p < 0.05; Fig. 3C). Furthermore, tube formation assays revealed that compared with the control, both the P10F and P20F scaffolds markedly improved the angiogenic potential of HUVECs, as evidenced by a significant increase in total tube length and the number of branch points (Fig. 3B). The wound-healing assay (Fig. S1B) demonstrated that after 24 h, the migration distance of HUVECs was notably greater in P20F and P30F groups compared to PLGA and blank groups.

The expression of the angiogenesis-related proteins VEGFR2 and HIF-1α was subsequently assessed via western blotting (Fig. 3E). The results demonstrated a significant upregulation of VEGFR2 and HIF-1α protein levels following treatment with P20F scaffolds.

### 3.4 PLGA/Fe_2_O_3_ scaffolds promote osteogenesis in BMSCs

ALP activity, an essential early marker of osteogenic differentiation, was quantified on Days 7 and 14 (Fig. 3D, right panel). ALP activity was significantly greater in the P20F group than in the blank, PLGA, and P10F groups. These observations are consistent with the results of Alizarin Red (AR) staining, which revealed increased calcium deposition in the P20F group (Fig. 3D, left panel). Quantitative analysis of ALP activity (Fig. S1D) showed that P10F, P20F, and P30F groups exhibited significantly higher ALP levels at Day 7 and Day 14 compared with blank and PLGA groups (*p* < 0.05∗ or *p* < 0.01∗∗), with the highest activity observed in P30F. Consistently, Alizarin Red S staining (Fig. S1E) revealed more intense and widespread mineral deposition in P20F and P30F groups.

The expression of the osteogenic transcription factor Runx2, which is critical for osteoblast differentiation and chondrocyte maturation, was subsequently evaluated by Western blot analysis. As shown in Fig. 3F, treatment with the 20 % IONPs PLGA/Fe_2_O_3_ scaffold markedly upregulated Runx2 protein expression.

Collectively, these findings suggest that compared with the 10 % IONPs formulation, the PLGA/Fe_2_O_3_ scaffold promotes osteogenic differentiation, with the 20 % IONPs formulation exhibiting superior performance. Since P20F exhibited bioactivity comparable to P30F while maintaining superior structural stability, it was selected for subsequent experiments. This observation aligns with previous reports indicating that moderate incorporation of magnetic nanoparticles (≤20 % w/w) into polymeric scaffolds provides optimal stimulation of osteogenesis [[30], [31], [32]].

### 3.5 PLGA/Fe_2_O_3_ scaffolds promote angiogenesis and osteogenesis via IONPs effects

To further clarify the role of Fe^3+^ and IONPs, we prepared FeCl_3_ solutions at concentrations equivalent to the cumulative Fe^3+^ release measured at 6 (3 mg/L Fe^3+^) and 8 (16 mg/L Fe^3+^) weeks, as well as IONPs suspensions with the same molar Fe content. Interestingly, FeCl_3_ solutions at 16 mg/L concentration might exert cell toxicity to BMSC (Fig. S2C) and did not significantly promote osteogenesis or angiogenesis *in vitro*, whereas both IONPs suspensions and the P20F scaffolds markedly enhanced BMSC osteogenic differentiation by increased ALP activity (Fig. S2D), mineralization (Fig. S2E), and HUVEC proliferation and migration (Fig. S2F and G).

### 3.6 PLGA/Fe_2_O_3_ scaffolds promote bone regeneration and angiogenesis *in vivo*

#### 3.6.1 Micro-CT analysis

On the basis of the superior performance of the 20 % IONPs-containing scaffolds demonstrated in all the *in vitro* experiments, this group was selected for *in vivo* evaluation. PLGA/Fe_2_O_3_ scaffolds were implanted into a femoral condyle bone defect model in rabbits (Fig. 4A). No postoperative complications, such as anorexia, diarrhea, or infection, were observed in any of the animals. At 12 weeks post-surgery, femoral specimens were harvested and subjected to micro-CT scanning and 3D reconstruction to assess bone regeneration. Compared with the Blank (with static magnetic field applied) and PLGA groups, the P20F group exhibited markedly increased new bone formation within the defect site (Fig. 4B). Quantitative analysis revealed significantly greater bone volume fraction (BV/TV), trabecular number (Tb.N) and bone mineral density (BMD) and lower trabecular separation (Tb.Sp) in the P20F group, indicating that enhanced osteogenesis was promoted by the PLGA/Fe_2_O_3_ scaffold under a static magnetic field (SMF) (Fig. 4C–F). No significant differences were observed between the blank and PLGA groups, suggesting that PLGA alone did not contribute to bone regeneration.

Angiogenesis was also evaluated via micro-CT-based angiography and 3D reconstruction. Compared with the other groups, the P20F group exhibited increased vascularization within the defect region (Fig. 4B), which was further validated by quantitative analysis showing significantly greater vessel volume in the P20F group than in the blank and PLGA groups (Fig. 4G).

### 3.7 Immunohistochemical staining analysis

**Fig. 5:**
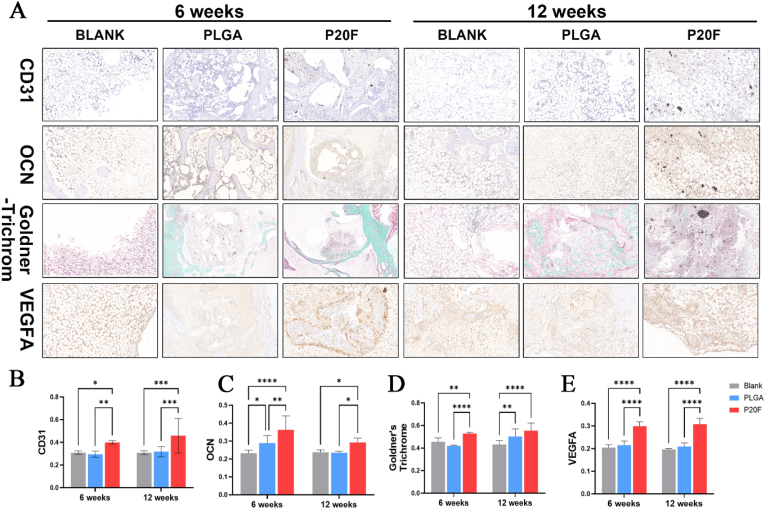
**Histological and immunohistochemical evaluation of angiogenesis and osteogenesis *in vivo* at 6 and 12 weeks post-implantation**. (A) Representative histological and immunohistochemical staining images of femoral defect sites implanted with blank, PLGA, or PLGA/20 %Fe_2_O_3_ (P20F) scaffolds. CD31 (endothelial marker) and VEGFA immunostaining indicated vascularization; OCN (osteocalcin) staining reflected osteoblast differentiation and bone matrix production; Goldner's trichrome staining showed mineralized bone tissue (green) and osteoids (red). At both time points, the P20F group exhibited stronger positive staining, indicating enhanced angiogenic and osteogenic responses. (B-E) Quantification of the mean optical density (MOD) of immunohistochemical staining for CD31, OCN, Goldner's trichrome, and VEGFA at 6 weeks (B) and 12 weeks. Compared with the PLGA and blank groups, the P20F group presented significantly higher expression of all the markers. The data are presented as the means ± SDs. Two-way ANOVA followed by Tukey's post-hoc test.∗P < 0.05, ∗∗P < 0.01, ∗∗∗P < 0.001. ∗∗∗∗P < 0.0001. (n = 6).

Immunohistochemical staining for osteocalcin (OCN), a key marker of osteoblast activity and bone formation, revealed the strongest positive expression in the P20F group after both 6 weeks and 12 weeks (Fig. 5A, B-E), further confirming enhanced osteogenic differentiation. Goldner's trichrome staining also revealed extensive new bone tissue formation in the P20F group, which is consistent with the micro-CT results. Immunohistochemical staining for key CD31 and VEGFA markers of endothelial cells and angiogenic activity revealed the highest expression levels in the P20F group, indicating that the PLGA/Fe_2_O_3_ scaffolds under SMF stimulation significantly promoted neovascularization *in vivo*.

### 3.8 Proteomic analysis of the osteogenesis and angiogenesis scaffolds

**Fig. 6:**
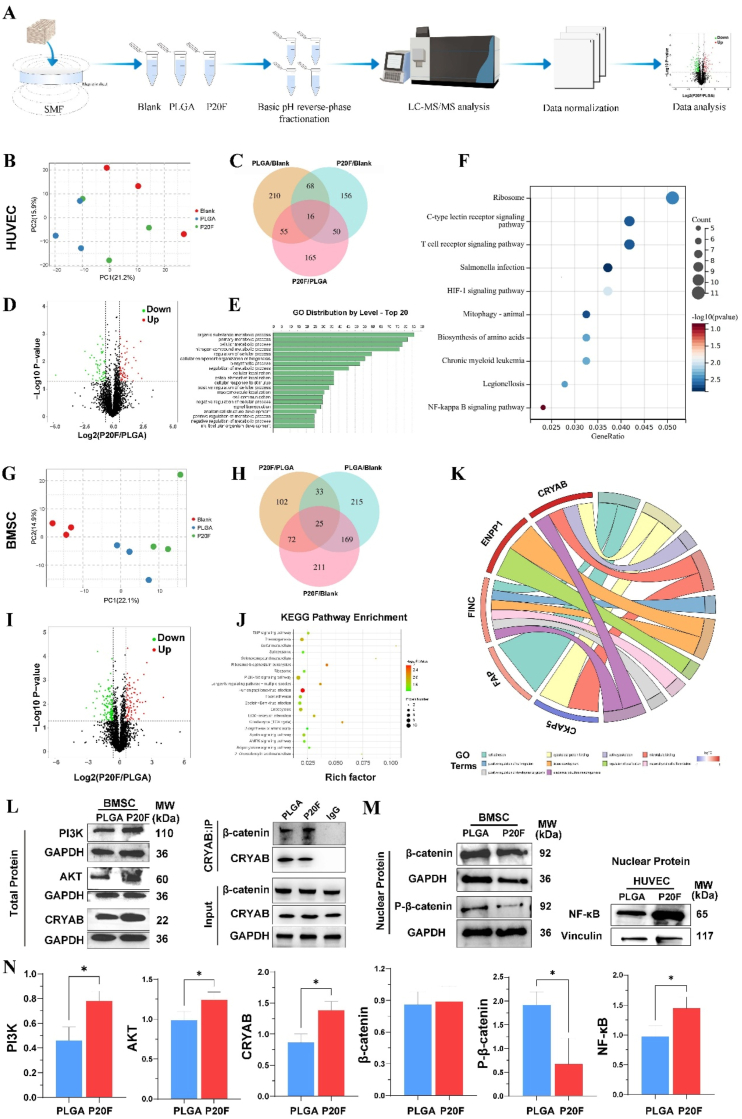
Proteomic analysis and mechanistic validation of angiogenic and osteogenic pathways activated by PLGA/20 %Fe2O3 scaffolds under static magnetic field (SMF) stimulation. (A) Workflow of the label-free quantitative proteomic analysis. HUVECs and BMSCs were cultured with blank, PLGA, or P20F scaffolds under SMF. Proteins were extracted, fractionated, analyzed by LC‒MS/MS, and processed for data normalization and bioinformatics analysis. (n = 3) (B, G) Principal component analysis (PCA) showing clear separation among sample groups in both HUVECs (B) and BMSCs (G). (C, H) Venn diagrams showing the number of differentially expressed proteins (DEPs) among different groups of HUVECs (C) and BMSCs (H). (D, I) Volcano plots depicting significantly upregulated (red) and downregulated (green) DEPs in P20F vs. PLGA groups in HUVECs (D) and BMSCs (I). (E, J) Gene Ontology (GO) enrichment (E) and Kyoto Encyclopedia of Genes and Genomes (KEGG) pathway enrichment analysis (J) highlighting biological processes and pathways associated with angiogenesis and osteogenesis. (F, K) KEGG pathway enrichment analysis showing key genes involved in Hif-1α and NF-κB signaling in HUVECs (F). Circular GO term‒protein interaction maps showing key enriched biological processes and representative DEPs in BMSCs (K), including CRYAB and other mechanistically relevant proteins. (L) Western blot analysis of PI3K, AKT, and CRYAB expression in BMSCs cultured with PLGA or P20F scaffolds (left panel). Co-IP anlysis of CRYAB and β-catenin in BMSCs(right panel) (M) Nuclear and cytoplasmic protein analysis in BMSCs and HUVECs. In the P20F group, nuclear β-catenin and NF-κB expression increased, and nuclear p-β-catenin expression decreased in the BMSCs, indicating pathway activation. ∗P < 0.05, ∗∗P < 0.01. (N) WB analysis using image J. Quantification revealed upregulation of PI3K/AKT signaling and NF-κB expressing in the P20F group. unpaired two-tailed Student's t-test ∗P < 0.05, ∗∗P < 0.01. (n = 3).

To further elucidate the molecular mechanisms underlying the osteogenic and angiogenic effects of the PLGA/Fe_2_O_3_ scaffolds under a static magnetic field, label-free quantitative proteomic analysis was performed (Fig. 6A). Principal component analysis (PCA) revealed clear separation among the blank, PLGA, and P20F groups, indicating distinct proteomic profiles in both HUVECs (Fig. 6B) and BMSCs (Fig. 6G).

Volcano plots demonstrated the distribution of differentially expressed proteins between the P20F and PLGA groups, with red and green dots indicating significantly upregulated and downregulated proteins, respectively (Fig. 6D and I). Venn diagrams further illustrated the overlap of differentially expressed genes among the comparison groups, with 16 common genes identified in HUVECs (Figs. 6C) and 25 in BMSCs (Fig. 6H).

In HUVECs, KEGG analysis indicated enrichment of the NF-κB and HIF-1α signaling pathways (Fig. 6F), both of which are associated with angiogenic activity. Chord diagrams further illustrate the associations between specific genes and enriched GO terms. As shown in Fig. 6F, we found that the genes were enriched in the Hif-1α and NF-Κb signaling pathways, which are strongly related to angiogenesis. In addition, angiogenesis-related GO terms included “nitrogen compound metabolic process”, “cellular response to stimulus” and “cell communication”.

GO enrichment analysis revealed that these common genes were significantly enriched in biological processes related to osteogenesis, including “PI3K-Akt signaling”, “anatomical structure morphogenesis,” “cytoskeletal protein binding,” and “microtubule binding” (Fig. 6J). As shown in the circular GO term–protein interaction map (**Figure** **6K**), CRYAB was significantly enriched in multiple biological processes related to osteogenesis, including cell adhesion, positive regulation of cell migration, actin cytoskeleton organization, and regulation of ossification. Moreover, quantitative proteomic analysis demonstrated that CRYAB expression in the P20F group was markedly elevated—approximately 2.7-fold higher than blank (p = 0.003) and 1.7-fold higher than PLGA (p = 0.02)—suggesting its involvement in the scaffold-induced biological response. KEGG pathway enrichment analysis of BMSCs revealed the involvement of genes that are differentially expressed in the PI3K-Akt signaling pathway, a known regulator of osteoblast differentiation and survival.

These findings suggest that the enhanced osteogenic and angiogenic effects observed with P20F scaffolds may be attributed to the activation of key molecular pathways and regulatory genes in both endothelial and mesenchymal stem cells.

### 3.9 CRYAB regulates BMSC osteogenesis via the PI3K-AKT signaling pathway

To further elucidate the molecular mechanism underlying CRYAB-mediated osteogenesis in BMSCs, key proteins involved in the PI3K-AKT signaling pathway were examined using western blotting on the basis of findings from proteomic analysis.

As shown in Fig. 6 L (left panel) the total protein levels of PI3K, AKT, and CRYAB were significantly greater in the P20F group than in the PLGA group. These results are consistent with previous findings suggesting that the activation of CRYAB together with the activity of the PI3K-AKT signaling pathway may promote osteogenic differentiation.

**Fig. 7:**
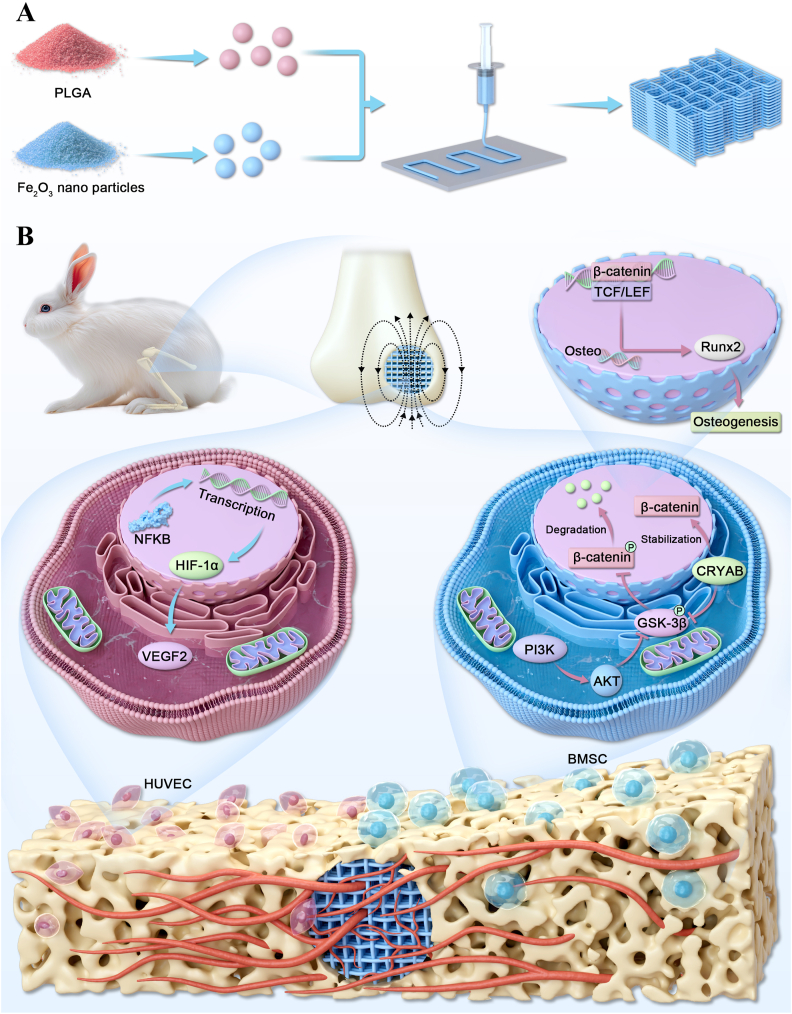
**Schematic illustration of scaffold fabrication and the proposed mechanism of osteogenesis and angiogenesis induced by PLGA/Fe_2_O_3_ scaffolds under static magnetic field stimulation**. (A) Fabrication of 3D-printed magnetic scaffolds. PLGA and Fe_2_O_3_ nanoparticles are mixed and processed via low-temperature extrusion-based 3D printing to form porous composite scaffolds. (B) Under static magnetic field (SMF) stimulation, the implanted PLGA/Fe_2_O_3_ scaffolds enhanced both angiogenesis and osteogenesis in the bone defect region. In HUVECs, magnetic scaffolds upregulated NF-κB and HIF-1α expression, leading to increased VEGF secretion and the promotion of angiogenesis. In BMSCs, SMF activated the PI3K/AKT pathway and increased CRYAB expression, which stabilized β-catenin by inhibiting its degradation via GSK-3β phosphorylation. Stabilized β-catenin translocates into the nucleus, promotes RUNX2 expression, and initiates osteogenic differentiation.

Mechanistically, activated AKT phosphorylates GSK-3β at the Ser9 residue, leading to its inactivation. This inactivation prevents GSK-3β from phosphorylating β-catenin, thereby preventing its ubiquitination and proteasomal degradation. As a result, β-catenin accumulates in the cytoplasm and subsequently translocates into the nucleus, where it activates the transcription of osteogenesis-related genes, such as RUNX2. (Fig. 6M left panel, Fig. 3F). In addition, P20F stimulation significantly increased the amount of β-catenin co-precipitated with CRYAB, providing direct evidence of CRYAB–β-catenin binding in response to P20F treatment (Fig. 6L right panel). Together, these findings suggest that activation of the PI3K-AKT pathway and CRYAB can promote osteogenesis by directly enhancing β-catenin stability (Fig. 7).

### 3.10 NF-κB regulates HUVEC angiogenesis via Hif-1α signaling

The Hif-1α signaling pathway is a central regulator of angiogenesis in human umbilical vein endothelial cells (HUVECs), particularly under hypoxic or stress conditions. Previous studies have shown that NF-κB can transcriptionally activate Hif-1α expression, thereby enhancing downstream angiogenic processes [33] According to our proteomic pathway enrichment analysis, several biological processes, such as “cellular response to stimulus” and “signal transduction”, were significantly enriched in the P20F group, suggesting the involvement of hypoxia-responsive pathways.

To experimentally validate whether NF-κB regulates angiogenesis through Hif-1α, we examined both NF-κB and Hif-1α protein levels. As shown in Fig. 3E, Hif-1α expression was significantly upregulated in the P20F group. Concurrently, Western blot analysis revealed increased nuclear NF-κB expression (Fig. 6M, right panel). These findings, together with those of previous reports and the known NF-κB–Hif-1α regulatory axis, support the hypothesis that P20F treatment promotes angiogenesis through activation of the NF-κB/Hif-1α signaling pathway in HUVECs (Fig. 7).

## 4 Discussion

The application of tissue engineering scaffolds in bone regeneration has received increasing attention. An ideal scaffold should possess excellent biocompatibility, controllable degradation, tunable porosity, and the ability to stimulate cell activity and differentiation [29]. However, a common challenge is the limited cell infiltration into the inner regions of three-dimensional scaffolds, often resulting in central tissue necrosis due to poor vascularization and nutrient diffusion [34]. The coupling of osteogenesis and angiogenesis plays a pivotal role in effective bone regeneration, as adequate vascularization is essential for nutrient transport, waste removal, and osteoprogenitor cell recruitment during bone healing. Conversely, bone-forming cells secrete growth factors (such as VEGF) that attract and stimulate endothelial cells, while endothelial cells in turn release “angiocrine” signals (e.g. BMP-2/4) that promote osteoblast differentiation [35].

The integration of iron oxide nanoparticles (IONPs) into tissue engineering scaffolds has evolved rapidly in recent years, with growing attention to how the assembly strategy affects both the magnetic response and the biological outcome, such as osteogenesis and angiogenesis. [19,36]. IONPs can be incorporated through bulk blending during scaffold fabrication, chemical conjugation, or by spatially patterned deposition to create magnetic gradients [[37], [38], [39]]. Among these, 3D printing–assisted approaches enable precise control of pore geometry and particle distribution, allowing reproducible modulation of local magnetic microenvironments [[40], [41], [42]].

Mechanistically, IONPs are now recognized to influence bone regeneration through several complementary routes. Under static or dynamic magnetic fields, IONPs generate localized mechanical forces and microgradients that stimulate mechanosensitive ion channels, integrin clustering, and cytoskeletal reorganization [43], thereby activating osteogenesis-related signaling such as MAPK, BMP/Smads, and PI3K/AKT/β-catenin [44]. IONPs-loaded scaffolds may enhance angiogenesis, however, the mechanistic basis for this effect is not yet fully understood and warrants further investigation.

Our study expands on these findings by providing micro-CT and vascular reconstruction data as concurrent 3D evidence of coupled regeneration: higher BV/TV and improved trabecular microarchitecture (Tb.N and BMD increase, Tb.Sp decrease) were matched by increased vessel volume fraction by P20F under SMF. Moreover, 3D confocal reconstructions demonstrated that viable cells penetrate into interior scaffold pores with clustering along struts. These dual lines of spatial evidence support the notion that scaffold design and magneto-stimulation do not simply boost individual endpoints but reinforce osteogenesis–angiogenesis coupling via coordinated microenvironmental control.

Moreover, Proteomic tool was used to screen CRYAB as among the most upregulated proteins in the BMSC cultured with P20F + SMF condition. Co-immunoprecipitation confirmed that CRYAB forms complexes with β-catenin more abundantly under P20F, correlating with enhanced PI3K/AKT signaling, increased RUNX2 expression, and suppressed nuclear p-β-catenin. This positions CRYAB upstream as a stabilizer or chaperone of β-catenin, promoting its nuclear localization and downstream osteogenic transcription. Recent studies reinforce CRYAB's emerging role in skeletal biology. Study has showed that CRYAB suppresses ferroptosis, stabilizes redox balance, and supports osteogenic differentiation via FTH1 regulation [45]. A review has outlined CRYAB's role as a stress-responsive chaperone and its implications in bone homeostasis and pathology [46]. These connections strengthen CRYAB as a logical mechanistic focus and not a post-hoc choice. Zhu et al. demonstrated that CRYAB promotes osteogenic differentiation of human BMSCs by physically interacting with β-catenin, protecting it from ubiquitination and degradation, thereby stabilizing canonical Wnt/β-catenin signaling [47]. Increased expression of CRYAB facilitates the nuclear accumulation of β-catenin, a core effector of the PI3K/AKT signaling pathway, which has been demonstrated to be critical for osteogenic differentiation of human BMSCs [47].

Bone regeneration is a highly orchestrated process that requires simultaneous new bone formation (osteogenesis) and new blood vessel formation (angiogenesis). These two processes are biologically interdependent and tightly coupled [48]. Thus, effective bone repair requires a coordinated enhancement of both vascular ingrowth and new bone formation, often referred to as osteogenic-angiogenic coupling [35]. In our study, The concurrent activation of the CRYAB/PI3K-AKT pathway in BMSCs and the NF-κB/HIF-1α pathway in endothelial cells by our suggests a synergistic crosstalk between bone formation and vessel formation within the scaffold. Osteogenic cells and endothelial cells exchange molecular signals that reinforce each other's activity. Specifically, as BMSCs differentiate into osteoblasts (a process enhanced by PI3K/AKT signaling), they secrete vascular endothelial growth factor (VEGF) and other angiogenic cytokines into the local milieu. The elevated VEGF can bind to receptors on nearby HUVECs, further stimulating their angiogenic response and possibly amplifying HIF-1α activity in a positive feedback manner. Conversely, activated endothelial cells (with NF-κB/HIF-1α signaling) not only form perfused capillaries but also release osteogenic factors-the so-called angiocrine factors [35]. The PI3K–AKT and HIF-1α pathways may also intersect at certain nodes of cell signaling. AKT activation can enhance HIF-1α signaling (through increased translation and stability of HIF-1α protein), and HIF-1α can in turn promote AKT/mTOR activity as shown in coupling studies [35,49]. Meanwhile, NF-κB activity in inflammatory environments could potentially stimulate PI3K–AKT in mesenchymal cells, as NF-κB target genes include various growth factors and cytokines. Immune cells such as macrophages can also influence vascularization and bone formation through the secretion of cytokines and growth factors that modulate endothelial cell activity and osteogenic differentiation. Although the precise molecular intersections in our system require further investigation, it is clear that CRYAB/PI3K–AKT-driven osteogenesis and NF-κB/HIF-1α-driven angiogenesis act in concert, each providing signals that bolster the other process. This bidirectional crosstalk accelerates the overall bone repair process beyond what either pathway could achieve alone. Future work could further systematically investigating the immune response, including macrophage polarization dynamics, in our Fe_2_O_3_/PLGA scaffold system.

Another strength of our study lies in rigorously discriminating between soluble iron effects and those deriving from embedded nanoparticles. Although scaffolds gradually release Fe^3+^ during degradation, we found that equivalent FeCl_3_ solutions — particularly at higher concentrations — did not reproduce the osteogenic or angiogenic enhancements and even induced mild cytotoxicity. In contrast, Fe_2_O_3_ nanoparticle suspensions and scaffolds itself robustly promoted ALP activity, mineralization, and endothelial proliferation and migration. This evidence supports that the magnetic nanoparticle-mediated microenvironment-rather than free ions-drives the observed biological benefits. This is consistent with recent mechano-genetic paradigms in magnetic scaffold research, which emphasize the importance of spatially localized magnetic cues rather than bulk ion release.

Together, these results highlight that the therapeutic benefits of magnetic scaffolds are not merely attributable to iron ion release but rather to the magneto-mechanical microenvironment orchestrated by IONPs, offering opportunities to fine-tune scaffold design and magnetic stimulation for more effective bone regeneration.

## 5 Conclusions

In this study, we fabricated a 3D-printed PLGA scaffold incorporating Fe_2_O_3_ nanoparticles and confirmed its biosafety *in vitro* and *in vivo*. Micro-CT combined with vascular contrast enabled direct 3D visualization of bone regeneration and neovascularization, demonstrating the scaffold's dual osteogenic and angiogenic potential. Proteomic and pathway analyses revealed that the scaffold promotes osteogenesis by upregulating CRYAB, which stabilizes β-catenin and activates PI3K/AKT signaling. These findings highlight CRYAB as a promising mechanistic target and provide new insight into the coupling of bone and vessel formation. Future studies focusing on long-term implantation, load-bearing models, and dynamic osteo-angiogenic crosstalk will further clarify its translational potential.

## CRediT authorship contribution statement

**Jieying Liu:** Writing – original draft, Visualization, Validation, Project administration, Methodology, Data curation, Conceptualization. **Fuze Liu:** Writing – original draft, Visualization, Validation, Project administration, Formal analysis, Data curation. **Cairong Li:** Writing – original draft, Visualization, Supervision, Project administration, Funding acquisition, Formal analysis. **Zhengyao Li:** Project administration, Methodology. **Tianle Li:** Visualization, Supervision. **Yuanhao Wu:** Methodology, Formal analysis. **Di Wu:** Project administration, Methodology, Formal analysis, Data curation. **Yue Huang:** Project administration. **Hui Chen:** Supervision, Project administration, Methodology, Formal analysis, Data curation. **Hai Wang:** Writing – review & editing, Validation, Supervision, Methodology, Funding acquisition, Formal analysis, Data curation, Conceptualization. **Yuxiao Lai:** Writing – review & editing, Supervision, Funding acquisition, Data curation, Conceptualization. **Zhihong Wu:** Writing – review & editing, Supervision, Investigation, Funding acquisition, Data curation, Conceptualization.

## Ethics approval and consent to participate

The procedures of all animal experiments were approved by the ethics committee of the Peking Union Medical College Hospital(Y0638). The experiments was conducted in accordance with the Guide for the Care and Use of Laboratory Animals (GB14925-2010; NIH), and the Laboratory Animal Center of Peking Union Medical College Hospital.

## Declaration of competing interest

The authors have declared that no competing interest exists.
