# Supplementary material for: 3D-printed magnetic scaffolds promote bone and vessel regeneration through CRYAB/PI3K-AKT and NF-κB pathways identified by proteomics
Source: Bioact Mater. 2025 Oct 23;56:277–93. doi: 10.1016/j.bioactmat.2025.10.013 (PMC12593645; doi:10.1016/j.bioactmat.2025.10.013)
Supplement: Multimedia component 2 [file mmc2.docx]

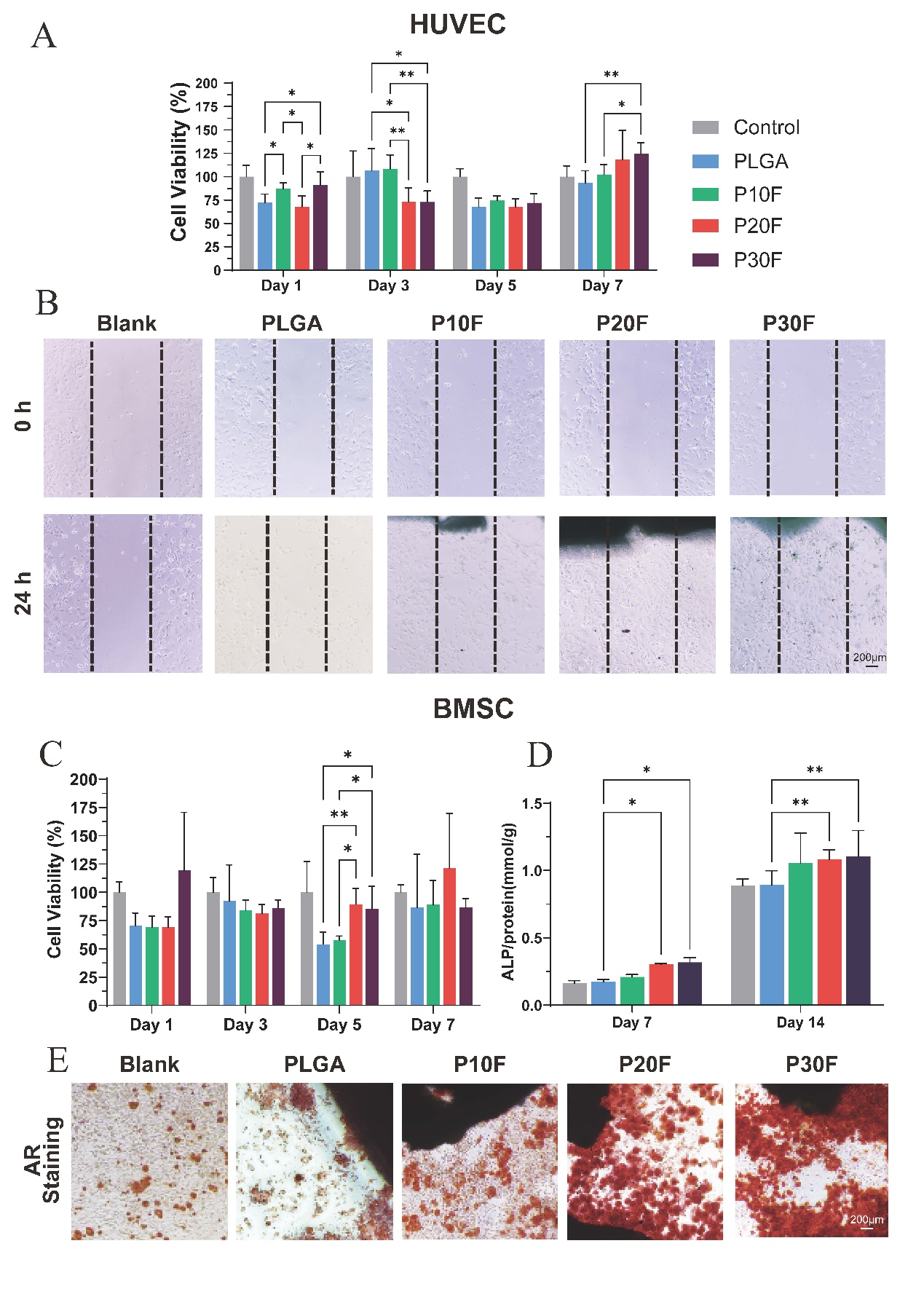


**Figure S1. In vitro evaluation of cell viability, migration, and osteogenic differentiation on PLGA/Fe₂O₃ scaffolds under SMF.**

(A) HUVEC viability measured by CCK-8 assay at days 1, 3, 5, and 7 after co-culture with blank control, PLGA scaffold, or PLGA scaffolds containing 10%, 20%, or 30% Fe₂O₃ nanoparticles (P10F,P20F,P30F). Data are expressed as percentage relative to control.(n≥5)

(B) Scratch wound assay showing representative images of HUVEC migration at 0 h and 24 h for each group. Dashed lines mark the initial wound edges. Scale bar: 200 μm.

(C) BMSC viability measured by CCK-8 assay at days 1, 3, 5, and 7 under identical group conditions.

(D) ALP activity of BMSCs quantified at day 7 and day 14 and normalized to total protein content. (n≥5)

(E) Alizarin Red S staining of mineralized matrix deposition by BMSCs after 21 days of osteogenic induction. Representative images are shown for each group; mineralized nodules appear as red deposits. Scale bar: 200 μm.

Data are presented as mean ± SD (n ≥ 5 per group). Statistical significance was determined using two-way ANOVA followed by Tukey’s post-hoc test. *p < 0.05, **p < 0.01, ***p < 0.001 compared with indicated groups.


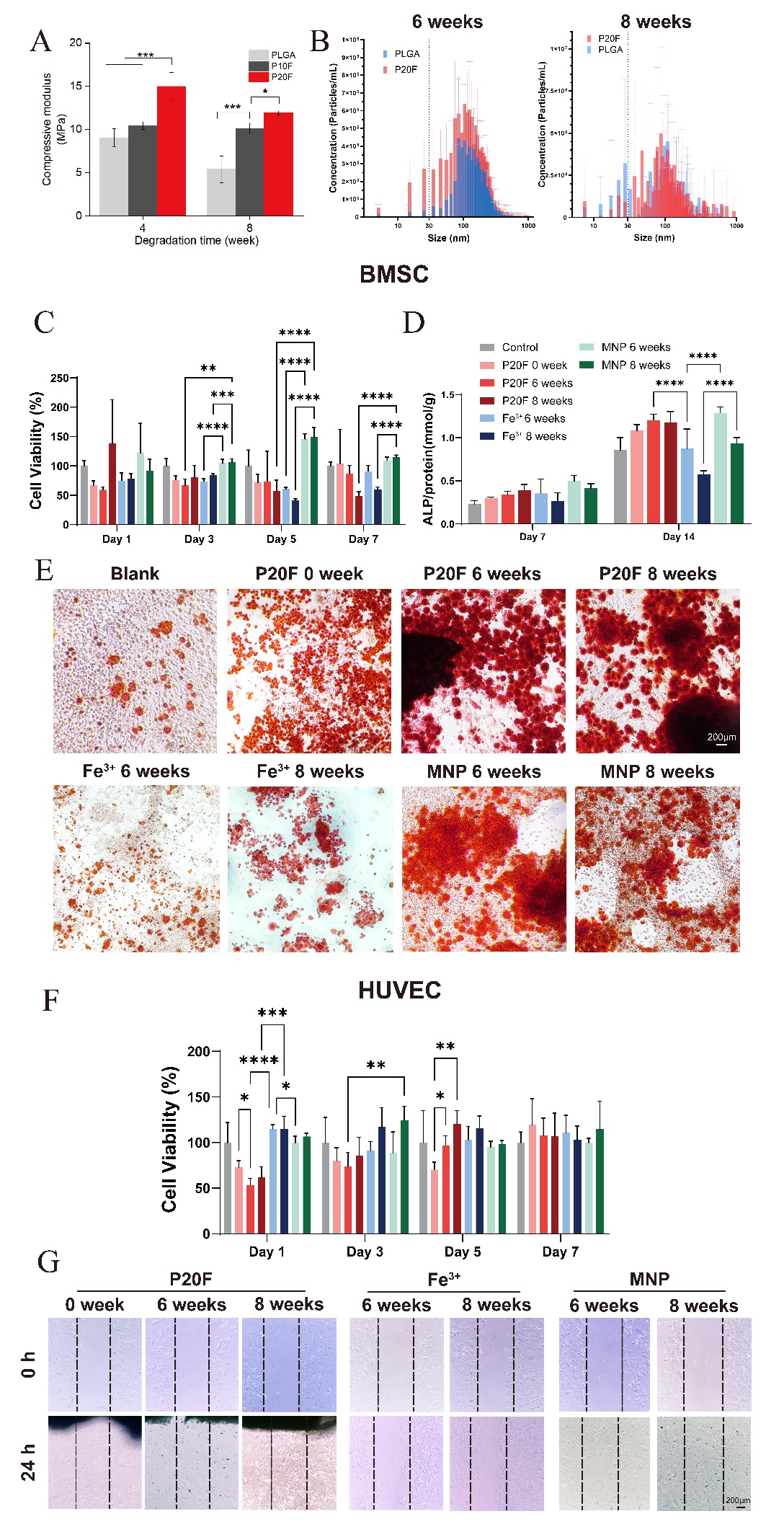


**Figure S2. Characterization of scaffold degradation, Fe³⁺ release equivalence, and their effects on BMSC osteogenesis and HUVEC function.**

(A) Compressive modulus of PLGA, P10F, and P20F scaffolds measured at 4 and 8 weeks of in vitro degradation, showing superior mechanical retention of P20F scaffolds.

(B) Nanoparticle tracking analysis (NTA) of scaffold extracts at 6 and 8 weeks. No detectable Fe₂O₃ nanoparticles were observed, confirming absence of particle release. (n≥5)

(C) BMSC viability assessed by CCK-8 assay at days 1, 3, 5, and 7 following exposure to extracts from P20F scaffolds (0, 6, and 8 weeks), Fe³⁺ solutions at equivalent cumulative release concentrations (6 and 8 weeks), and MNP suspensions with equivalent Fe content (6 and 8 weeks). (n≥5)

(D) ALP activity of BMSCs measured at day 7 and day 14 under the same treatment conditions, normalized to total protein. (n≥5)

(E) Alizarin Red S staining showing mineralized nodule formation after 21 days of osteogenic induction under indicated treatments. Mineral deposition is markedly enhanced in the P20F and MNP groups compared with Fe³⁺ groups. Scale bar: 200 μm.

(F) HUVEC viability evaluated by CCK-8 assay at days 1, 3, 5, and 7 under identical treatment conditions. (n≥5)

(G) Scratch wound assay depicting HUVEC migration at 0 h and 24 h for each treatment group. Dashed lines represent initial wound edges. Scale bar: 200 μm.

All data are presented as mean ± SD (n ≥ 5 per group). Statistical significance was determined by two-way ANOVA followed by Tukey’s post-hoc test. *p < 0.05, **p < 0.01, ***p < 0.001, ****p < 0.0001 between indicated groups.
